# Supplementary material for: Resistance to Chemotherapeutic 5-Fluorouracil Conferred by Modulation of Heterochromatic Integrity through Ino80 Function in Fission Yeast
Source: Int J Mol Sci. 2023 Jun 26;24(13):10687. doi: 10.3390/ijms241310687 (PMC10341484; doi:10.3390/ijms241310687)
Supplement: Supplementary file 1 [file ijms-24-10687-s001.zip › ijms-2443342-supplementary (with supplementary tables).docx]

Supplementary Figures


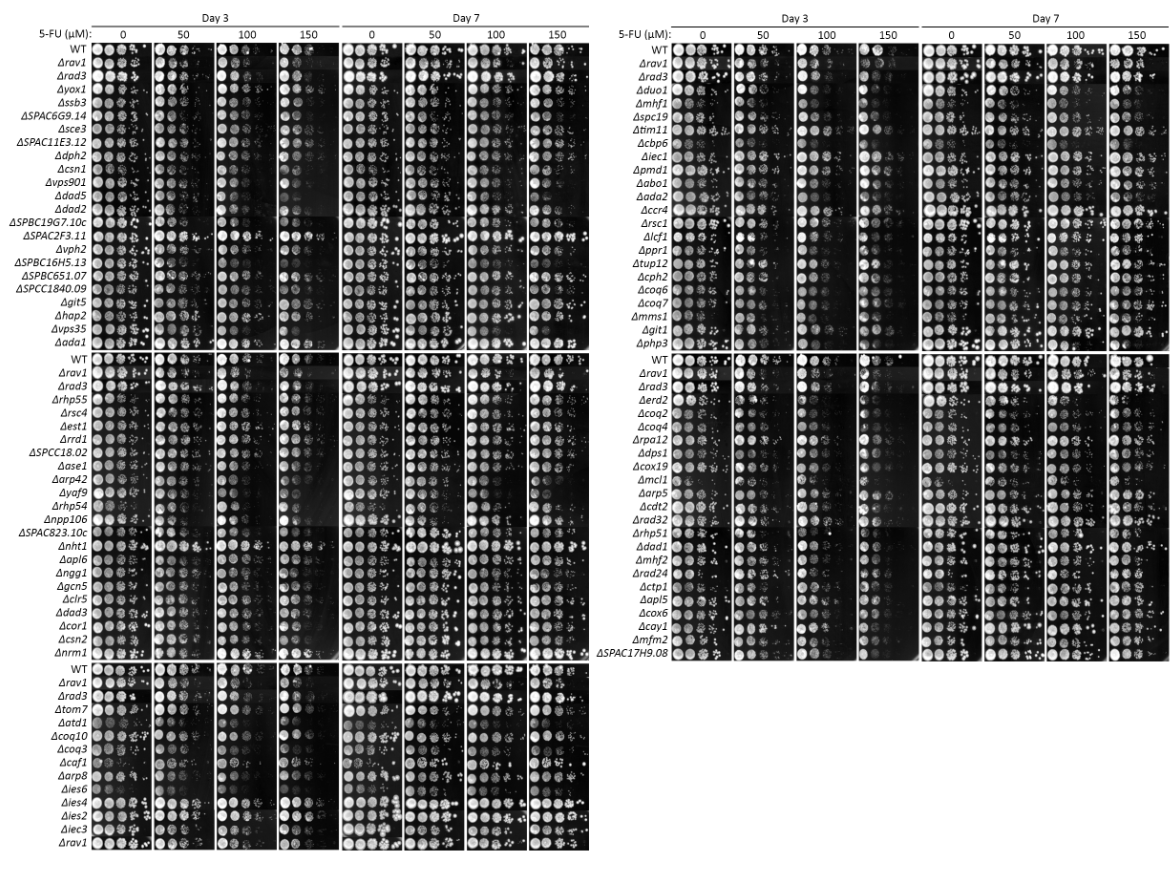


**Figure S1.** Prototrophic MER haploid deletion strains show different degree of hypersensitivity to 5-FU. Exponentially growing prototrophic MER strains were ten-fold serially diluted and individually spotted onto YEA plates incorporated with 0 (untreated), 50, 100 and 150 µM 5-FU and documented on day 3 and 7 after drug exposure.


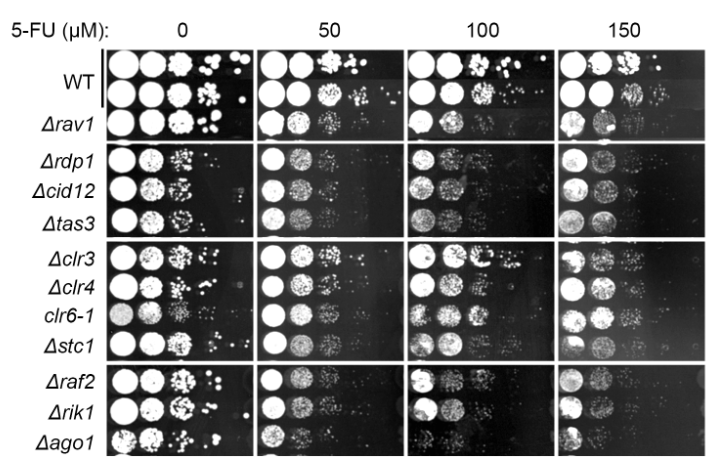


Figure S2. 5-FU hypersensitivity of strains with deletion of different genes function in heterochromatin formation. All strains are deletion mutant except *clr6-1*. Exponentially growing strains were ten-fold serially diluted before spotting onto plates incorporated with 0 (untreated), 50, 100 and 150 µM 5-FU and documented on day 3 and 7 after drug exposure. WT and *Δrav1* are negative and positive controls respectively.


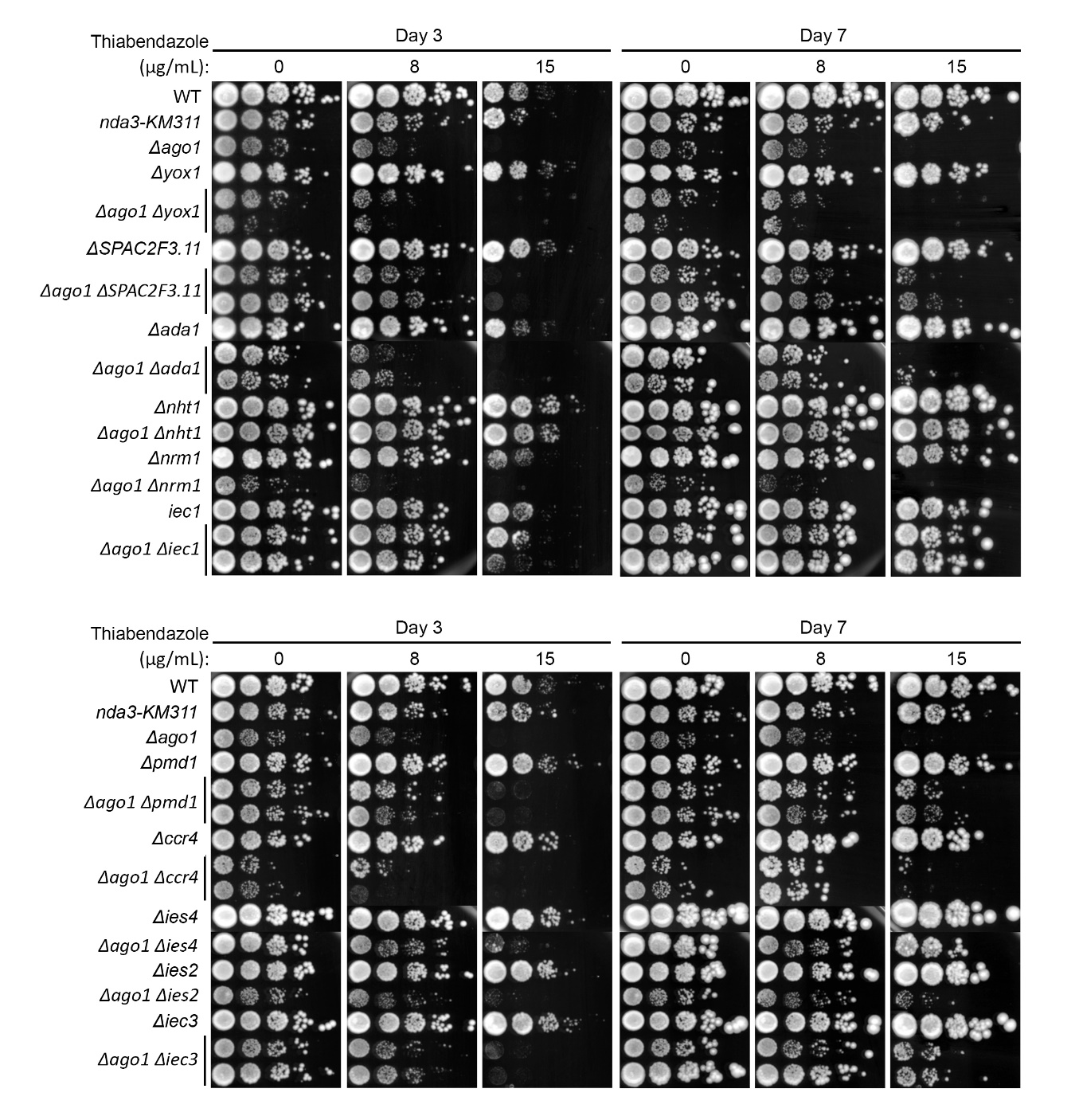


**Figure S3.** Genetic interaction of Ago1 with MER mutants that showed 5-FU resistance were tested on TBZ. TBZ hypersensitivity of the MER single and double mutants in combination with *Δago1* were tested at 0 (untreated), 8 and 15 µg/ml TBZ and documented on day 3 and 7 upon drug exposure.


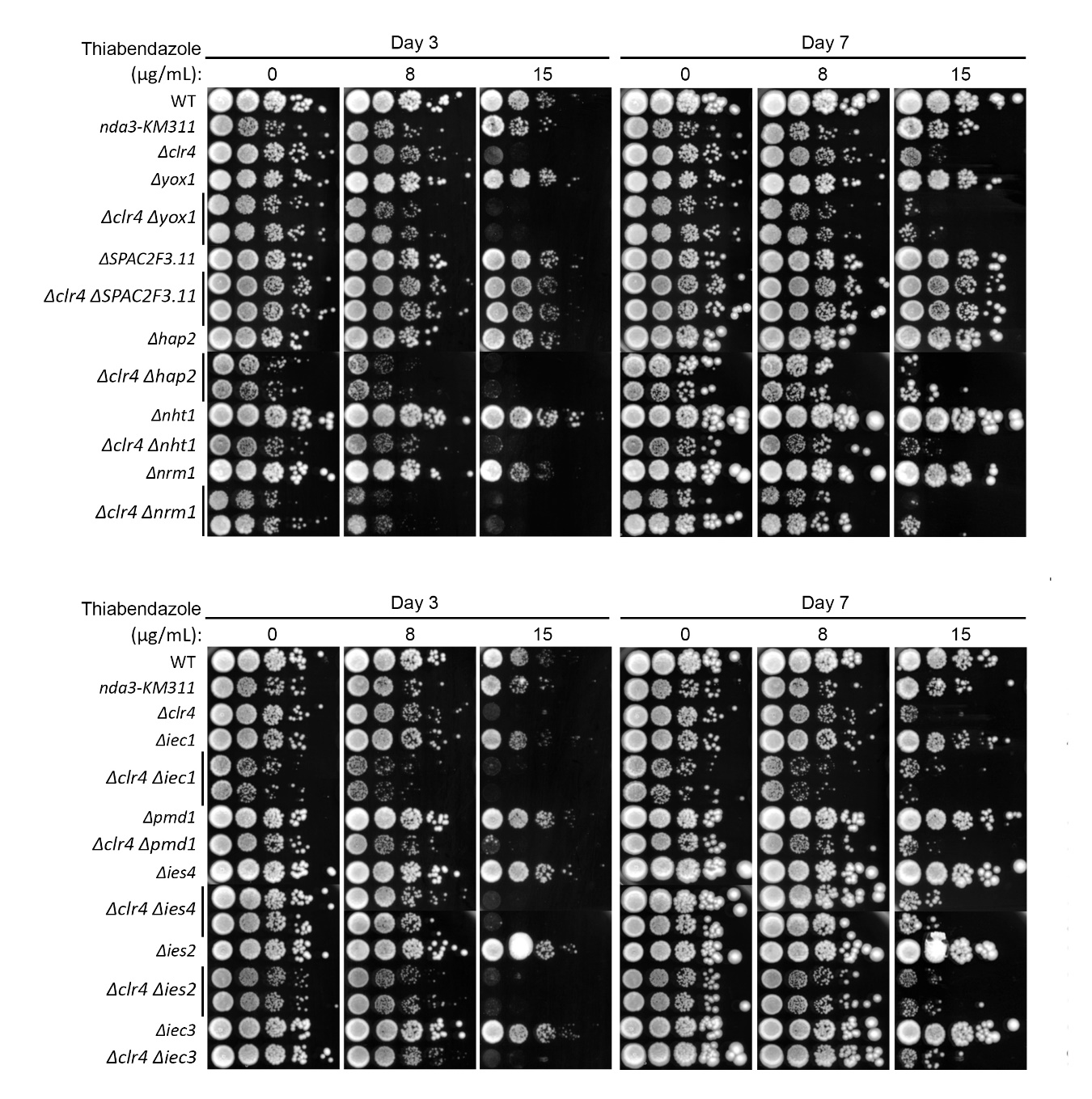


**Figure S4.** Genetic interaction of Clr4 with 5-FU-resistant MER mutants were tested on TBZ. TBZ hypersensitivity of the MER single and double mutants in combination with *Δclr4* were tested at 0 (untreated), 8 and 15 µg/ml TBZ and documented on day 3 and 7 upon drug exposure.

**Supplementary Tables**

**Table S1.** Mean S-score of MER genes on 5-FU with information on gene description and compound formation.

| **Gene** | **Group** | **Gene description** | **Complex formation** | **Mean S-score** | |
| --- | --- | --- | --- | --- | --- |
|  |  |  |  | **Day 3** | **Day 7** |
| *Δabo1* | Chromatin remodelling | Histone H3-H4 chaperone (ATP-dependent) Abo1 | - | -0.26 | 0.08 |
| *Δarp42* |  | SWI/SNF and RSC complex subunit Arp42 | SWI/SNF and RSC | -1.26 | -1.52 |
| *Δarp5* |  | Ino80 complex actin-like protein Arp5 | Ino80 | -0.22 | -0.20 |
| *Δarp8* |  | Ino80 complex actin-like protein Arp8 | Ino80 | -0.62 | -0.16 |
| *Δclr5* |  | Clr5 protein | - | -0.17 | 0.00 |
| *Δcph2* |  | Clr6 histone deacetylase associated PHD-finger protein Cph2 | Rpd3S | -0.43 | -0.16 |
| *Δest1* |  | Telomerase regulator Est1 | Telomerase holoenzyme | 0.08 | -0.30 |
| *Δiec1* |  | Ino80 complex subunit Iec1 | Ino80 | 0.49 | 0.16 |
| *Δiec3* |  | Ino80 complex subunit Iec3 | Ino80 | 0.46 | 0.23 |
| *Δies2* |  | Ino80 complex subunit Ies2 | Ino80 | 0.52 | 0.23 |
| *Δies4* |  | Ino80 complex subunit Ies4 | Ino80 | 0.52 | 0.23 |
| *Δies6* |  | Ino80 complex subunit Ies6 | Ino80 | -0.31 | -0.27 |
| *Δnht1* |  | Ino80 complex HMG box subunit Nht1 | Ino80 | 0.31 | -0.03 |
| *Δrsc1* |  | RSC complex subunit Rsc1 | RSC-Type | -0.74 | -0.39 |
| *Δrsc4* |  | RSC complex subunit Rsc4 | RSC-Type | -0.18 | -0.58 |
| *ΔSPCC16C4.20c* |  | Ino80 complex, HMG box protein Hap2 | Ino80 | 0.31 | -0.03 |
| *Δyaf9* |  | NuA4 and Swr1 complex YEATS family subunit Yaf9 | NuA4 / Swr1 | -1.40 | -2.05 |
| *Δase1* | Chromosome segregation | Antiparallel microtubule cross-linking factor Ase1 | - | -0.73 | -0.47 |
| *Δdad1* |  | DASH complex subunit Dad1 | DASH | -1.11 | -0.35 |
| *Δdad2* |  | DASH complex subunit Dad2 | DASH | -0.89 | -0.42 |
| *Δdad3* |  | DASH complex subunit Dad3 | DASH | -0.54 | -0.16 |
| *Δdad5* |  | DASH complex subunit Dad5 | DASH | -1.40 | -1.33 |
| *Δduo1* |  | DASH complex subunit Duo1 | DASH | -0.70 | -0.76 |
| *Δmcl1* |  | DNA polymerase alpha accessory factor Mcl1 | - | -0.13 | -0.42 |
| *Δspc19* |  | DASH complex subunit Spc19 | DASH | -0.41 | -0.34 |
| *Δrad24* | DNA damage checkpoint | 14-3-3 protein Rad24 | - | -0.26 | -0.34 |
| *Δctp1* | DNA repair | CtIP-related endonuclease | - | -0.52 | -0.30 |
| *Δmhf1* |  | CENP-S ortholog, FANCM-MHF complex subunit Mhf1 | CENP-T-W-S-X / FANCM-MHF | -0.50 | -0.72 |
| *Δmhf2* |  | CENP-X ortholog, FANCM-MHF complex subunit Mhf2 | CENP-T-W-S-X / FANCM-MHF | -0.92 | -0.30 |
| *Δmms1* |  | Cul8-RING ubiquitin ligase complex subunit Mms1 (predicted) | Cul8-RING ubiquitin ligase | -0.59 | 0.08 |
| *Δrad32* |  | Mre11 nuclease | Mre11 | -0.11 | -0.23 |
| *Δrhp51* |  | RecA family recombinase Rad51/Rhp51 | - | -1.03 | -0.52 |
| *Δrhp54* |  | DNA-dependent ATPase Rad54/Rhp54 | - | -0.79 | -0.19 |
| *Δrhp55* |  | RecA family ATPase Rad55/Rhp55 | Rad51B-Rad51C-Rad51D-XRCC2 complex / Rad51C-XRCC3 complex | -0.36 | -0.54 |
| *Δssb3* |  | DNA replication factor A subunit Ssb3 | DNA replication factor A | -0.26 | -0.39 |
| *Δada1* | dNTP metabolism | Adenosine deaminase Ada1 | - | 0.34 | 0.16 |
| *Δcaf1* |  | CCR4-Not complex CAF1 family ribonuclease subunit Caf1 | CCR4-NOT core | 0.07 | -0.07 |
| *Δccr4* |  | CCR4-Not complex 3'-5'-exoribonuclease subunit 6 | CCR4-NOT core | 0.44 | 0.23 |
| *Δcdt2* |  | WD repeat protein Cdt2 | Cul4-RING E3 ubiquitin ligase | -0.53 | -0.35 |
| *Δcsn1* |  | COP9/signalosome complex subunit Csn1 | COP9 signalosome | -0.80 | -0.71 |
| *Δcsn2* |  | COP9/signalosome complex subunit Csn2 | COP9 signalosome | -0.89 | -0.60 |
| *ΔSPAC2F3.11* |  | Exopolyphosphatase, prune Ppx1 (predicted) | - | 0.69 | 0.16 |
| *Δlcf1* | Lipid metabolism | Long-chain-fatty-acid-CoA ligase Lcf1 | - | -0.14 | -0.03 |
| *Δapl5* | Membrane transporter | AP-3 adaptor complex subunit Apl5 | AP-3 adaptor | -0.41 | -0.46 |
| *Δapl6* |  | AP-3 adaptor complex subunit Apl6 (predicted) | AP-3 adaptor | -0.11 | -0.11 |
| *Δcor1* |  | Cornichon family protein | - | -0.54 | -0.50 |
| *Δerd2* |  | HDEL receptor | - | -1.78 | -1.56 |
| *Δnpp106* |  | Nucleoporin Npp106 |  | -0.99 | -0.61 |
| *Δpmd1* |  | Leptomycin transmembrane transporter Pmd1 | - | 0.30 | 0.16 |
| *Δrav1* |  | RAVE complex subunit Rav1 | RAVE | -0.58 | -0.56 |
| *ΔSPCC18.02* |  | Transmembrane transporter (predicted) | - | -0.32 | -0.61 |
| *Δvph2* |  | Endoplasmic reticulum membrane protein involved in assembly of the V-ATPase (predicted) | - | -0.56 | -0.34 |
| *Δvps35* |  | Retromer complex subunit Vps35 | Retromer | -1.88 | -2.20 |
| *Δvps901* |  | Guanyl-nucleotide exchange factor Vps902 | - | -1.17 | -1.09 |
| *Δatd1* | Mitochondria related | Aldehyde dehydrogenase | - | -0.50 | -0.49 |
| *Δcbp6* |  | Mitochondrial Cob1 translation regulator Cbp6 (predicted) | Cbp3p-Cbp6 | -0.24 | -0.67 |
| *Δcoq10* |  | Mitochondrial ubiquinone binding protein Coq10 | - | -0.40 | -0.38 |
| *Δcoq2* |  | Para-hydroxybenzoate--polyprenyltransferase Ppt1 | - | -0.63 | -0.31 |
| *Δcoq3* |  | Hexaprenyldihydroxybenzoate methyltransferase Coq3 | - | -0.51 | -0.60 |
| *Δcoq4* |  | Ubiquinone biosynthesis protein Coq4 | - | -0.52 | -0.35 |
| *Δcoq6* |  | Monooxygenase Coq6 (predicted) | - | -0.89 | -0.16 |
| *Δcoq7* |  | Ubiquinone biosynthesis protein Coq7 | - | -0.84 | 0.16 |
| *Δcox19* |  | Mitochondrial copper chaperone for cytochrome c oxidase Cox19 (predicted) | - | -0.56 | -0.47 |
| *Δcox6* |  | Cytochrome c oxidase subunit VI | - | -0.71 | -0.20 |
| *Δdps1* |  | Decaprenyl diphosphate synthase subunit Dps1 | Decaprenyl diphosphate synthase | -0.47 | -0.34 |
| *Δppr1* |  | Mitochondrial PPR repeat protein Ppr1 | - | -0.67 | -0.44 |
| *ΔSPAC11E3.12* |  | Mitochondrial thioredoxin family protein, implicated in sulfur cluster assembly | - | -0.11 | -0.39 |
| *ΔSPAC17H9.08* |  | Mitochondrial carrier, coenzyme A | - | -1.16 | -0.31 |
| *ΔSPAC823.10c* |  | Mitochondrial carrier, glycine Hem25 (predicted) |  | -0.77 | -0.08 |
| *ΔSPCC1840.09* |  | Ubiquinone biosynthesis protein Coq11 | - | -0.22 | -0.23 |
| *Δtim11* |  | F1-FO ATP synthase subunit E | - | 0.16 | -0.16 |
| *Δtom7* |  | Mitochondrial TOM complex subunit Tom7 | Mitochondrial outer membrane translocase | -0.10 | -0.08 |
| *Δgit1* | Signal transduction | C2 domain protein Git1 | - | 0.19 | 0.23 |
| *Δgit5* |  | Heterotrimeric G protein beta (WD repeat) subunit Git5 | Heterotrimeric G-protein | -0.04 | 0.08 |
| *Δrrd1* |  | Protein phosphatase type 2A regulator, PTPA family Ypa1 | Protein phosphatase type 2A | -0.10 | -0.19 |
| *Δada2* | Transcription related | SAGA complex subunit Ada2 | SAGA | -0.73 | -0.74 |
| *Δgcn5* |  | SAGA complex histone acetyltransferase catalytic subunit Gcn5 | SAGA | -0.89 | -0.47 |
| *Δngg1* |  | SAGA complex subunit Ngg1/Ada3 | SAGA | -0.78 | -0.54 |
| *Δnrm1* |  | MBF complex corepressor Nrm1 | - | 0.31 | 0.08 |
| *Δphp3* |  | CCAAT-binding factor complex subunit Php3 | CCAAT-binding factor | -1.29 | -1.57 |
| *Δrpa12* |  | DNA-directed RNA polymerase complex I subunit Rpa12 | RNA polymerase I | 0.16 | -0.12 |
| *Δtup12* |  | Transcriptional corepressor Tup12 | - | -0.29 | -0.16 |
| *Δyox1* |  | MBF complex corepressor Yox1 | - | 0.69 | 0.00 |
| *Δdph2* | Translation related | Diphthamide biosynthesis protein Dph2 (predicted) | Protein-containing | -0.24 | -0.47 |
| *Δsce3* |  | Translation initiation factor (predicted) | - | -0.03 | -0.47 |
| *ΔSPAC6G9.14* |  | Pumilio family RNA-binding protein Puf4 (predicted) | - | -0.36 | -0.61 |
| *ΔSPBC19G7.10c* |  | Topoisomerase II-associated deadenylation-dependent mRNA-decapping factor Pdc2 (predicted) | - | -1.26 | -0.83 |
| *Δcay1* | Unknown sequence | Cactin, spliceosome complex subunit | Spliceosome | -0.48 | -0.30 |
| *Δmfm2* |  | M-factor precursor Mfm2 | - | -0.46 | -0.20 |
| *ΔSPBC16H5.13* |  | WD repeat protein, human WDR7 ortholog | - | -1.44 | -0.82 |
| *ΔSPBC651.07* |  | *Schizosaccharomyces* specific protein Mug166 | - | -0.11 | -0.19 |

The S-scores are the mean of three replicates.

**Table S2.** Extent of proliferation for the genetic interaction of Ago1 with 5-FU-resistant MER mutants tested on different concentration of 5-FU.

|  | **Day 3** | | | | |  | **Day 7** | | | |
| --- | --- | --- | --- | --- | --- | --- | --- | --- | --- | --- |
| **5-FU (μM)** | **0** | **50** | **100** | **150** |  | | **0** | **50** | **100** | **150** |
| WT | ++++ | ++++ | +++ | ++ |  | | ++++ | ++++ | ++++ | ++++ |
| *Δrav1* | ++++ | + | - | - |  | | ++++ | +++ | + | + |
| *Δago1* | +++ | - | - | - |  | | +++ | + | + | + |
| *Δyox1* | ++++ | ++++ | ++++ | +++ |  | | ++++ | ++++ | ++++ | ++++ |
| *Δago1 Δyox1* | +++ | - | - | - |  | | +++ | ++ | + | + |
| *Δago1 Δyox1* | +++ | + | - | - |  | | +++ | ++ | + | + |
| *ΔSPAC2F3.11* | ++++ | ++++ | ++++ | ++++ |  | | ++++ | ++++ | ++++ | ++++ |
| *Δago1 ΔSPAC2F3.11* | +++ | +++ | ++ | ++ |  | | +++ | +++ | +++ | +++ |
| *Δago1 ΔSPAC2F3.11* | +++ | +++ | ++ | ++ |  | | +++ | +++ | +++ | +++ |
| *Δada1* | ++++ | ++++ | ++++ | ++++ |  | | ++++ | ++++ | ++++ | ++++ |
| *Δago1 Δada1* | +++ | + | + | + |  | | +++ | +++ | +++ | +++ |
| *Δago1 Δada1* | +++ | + | + | + |  | | +++ | +++ | +++ | +++ |
| *Δnht1* | ++++ | ++++ | ++++ | ++++ |  | | ++++ | ++++ | ++++ | ++++ |
| *Δago1 Δnht1* | ++++ | +++ | +++ | ++ |  | | ++++ | +++ | +++ | +++ |
| *Δnrm1* | ++++ | ++++ | ++++ | +++ |  | | ++++ | ++++ | ++++ | +++ |
| *Δago1 Δnrm1* | ++ | - | - | - |  | | ++ | + | + | + |
|  |  |  |  |  |  | |  |  |  |  |
| WT | ++++ | ++++ | +++ | ++ |  | | ++++ | ++++ | ++++ | ++++ |
| *Δrav1* | ++++ | + | - | - |  | | ++++ | +++ | + | - |
| *Δago1* | +++ | - | - | - |  | | +++ | + | + | + |
| *Δiec1* | ++++ | ++++ | +++ | +++ |  | | ++++ | ++++ | ++++ | ++++ |
| *Δago1 Δiec1* | ++++ | +++ | +++ | ++ |  | | ++++ | +++ | +++ | +++ |
| *Δago1 Δiec1* | ++++ | +++ | +++ | ++ |  | | ++++ | +++ | +++ | +++ |
| *Δpmd1* | ++++ | ++++ | +++ | +++ |  | | ++++ | ++++ | ++++ | ++++ |
| *Δago1 Δpmd1* | ++++ | ++ | ++ | ++ |  | | ++++ | +++ | +++ | +++ |
| *Δago1 Δpmd1* | ++++ | ++ | ++ | ++ |  | | ++++ | +++ | +++ | +++ |
| *Δccr4* | ++++ | ++++ | +++ | +++ |  | | ++++ | ++++ | ++++ | ++++ |
| *Δago1 Δccr4* | +++ | + | + | - |  | | +++ | +++ | ++ | ++ |
| *Δago1 Δccr4* | +++ | + | + | - |  | | +++ | +++ | ++ | ++ |
| *Δies4* | ++++ | ++++ | ++++ | +++ |  | | ++++ | ++++ | ++++ | ++++ |
| *Δago1 Δies4* | ++++ | +++ | ++ | ++ |  | | ++++ | +++ | +++ | +++ |
| *Δies2* | ++++ | ++++ | +++ | +++ |  | | ++++ | ++++ | ++++ | ++++ |
| *Δago1 Δies2* | +++ | + | + | - |  | | ++++ | +++ | +++ | ++ |
| *Δiec3* | ++++ | ++++ | +++ | +++ |  | | ++++ | ++++ | ++++ | ++++ |
| *Δago1 Δiec3* | +++ | + | + | - |  | | ++++ | +++ | ++ | ++ |
| *Δago1 Δiec3* | +++ | + | + | - |  | | ++++ | +++ | ++ | ++ |

The extent of proliferation for each strain in Figure 2 was represented by: -, no growth, and increasing growth +, ++, +++, ++++.

**Table S3.** Extent of proliferation for the genetic interaction of Clr4 with 5-FU-resistant MER mutants tested on different concentration of 5-FU.

|  | **Day 3** | | | | |  | | **Day 7** | | | | |
| --- | --- | --- | --- | --- | --- | --- | --- | --- | --- | --- | --- | --- |
| **5-FU (μM)** | **0** | **50** | **100** | **150** |  | | **0** | | **50** | **100** | **150** |  |
| WT | ++++ | +++ | +++ | ++ |  | | ++++ | | ++++ | ++++ | ++++ |  |
| *Δrav1* | ++++ | + | - | - |  | | ++++ | | +++ | ++ | + |  |
| *Δclr4* | ++++ | + | - | - |  | | ++++ | | +++ | +++ | +++ |  |
| *Δyox1* | ++++ | ++++ | +++ | +++ |  | | ++++ | | ++++ | ++++ | ++++ |  |
| *Δclr4 Δyox1* | ++++ | + | + | + |  | | ++++ | | +++ | +++ | ++ |  |
| *Δclr4 Δyox1* | ++++ | ++ | + | + |  | | ++++ | | +++ | +++ | +++ |  |
| *ΔSPAC2F3.11* | ++++ | ++++ | +++ | +++ |  | | ++++ | | ++++ | ++++ | ++++ |  |
| *Δclr4 ΔSPAC2F3.11* | ++++ | ++ | ++ | ++ |  | | ++++ | | ++++ | ++++ | ++++ |  |
| *Δclr4 ΔSPAC2F3.11* | ++++ | ++ | ++ | ++ |  | | ++++ | | ++++ | ++++ | ++++ |  |
| *Δhap2* | ++++ | ++++ | +++ | +++ |  | | ++++ | | ++++ | ++++ | ++++ |  |
| *Δclr4 Δhap2* | +++ | - | - | - |  | | +++ | | +++ | - | - |  |
| *Δclr4 Δhap2* | ++ | - | - | - |  | | +++ | | +++ | - | - |  |
| *Δnht1* | ++++ | ++++ | ++++ | +++ |  | | ++++ | | ++++ | ++++ | ++++ |  |
| *Δclr4 Δnht1* | +++ | - | - | - |  | | +++ | | +++ | - | - |  |
| *Δnrm1* | ++++ | ++++ | +++ | +++ |  | | ++++ | | ++++ | ++++ | +++ |  |
| *Δclr4 Δnrm1* | +++ | - | - | - |  | | +++ | | ++ | - | - |  |
| *Δclr4 Δnrm1* | +++ | - | - | - |  | | +++ | | ++ | - | - |  |
| *Δiec1* | ++++ | ++++ | +++ | +++ |  | | ++++ | | ++++ | ++++ | ++++ |  |
| *Δclr4 Δiec1* | +++ | - | - | - |  | | +++ | | +++ | - | - |  |
| *Δclr4 Δiec1* | ++ | - | - | - |  | | +++ | | +++ | - | - |  |
|  |  |  |  |  |  | |  | |  |  |  |  |
| WT | ++++ | +++ | +++ | ++ |  | | ++++ | | ++++ | ++++ | ++++ |  |
| *Δrav1* | ++++ | ++ | - | - |  | | ++++ | | +++ | ++ | + |  |
| *Δclr4* | ++++ | ++ | - | - |  | | ++++ | | +++ | +++ | +++ |  |
| *Δpmd1* | ++++ | +++ | +++ | ++ |  | | ++++ | | ++++ | ++++ | ++++ |  |
| *Δclr4 Δpmd1* | ++++ | - | - | - |  | | ++++ | | +++ | +++ | +++ |  |
| *Δies4* | ++++ | ++++ | +++ | +++ |  | | ++++ | | ++++ | ++++ | ++++ |  |
| *Δclr4 Δies4* | ++++ | +++ | ++ | ++ |  | | ++++ | | ++++ | ++++ | ++++ |  |
| *Δclr4 Δies4* | ++++ | +++ | ++ | ++ |  | | ++++ | | ++++ | ++++ | ++++ |  |
| *Δies2* | ++++ | +++ | +++ | ++ |  | | ++++ | | ++++ | ++++ | ++++ |  |
| *Δclr4 Δies2* | ++++ | + | - | - |  | | ++++ | | ++++ | +++ | ++ |  |
| *Δclr4 Δies2* | ++++ | + | - | - |  | | ++++ | | ++++ | +++ | ++ |  |
| *Δiec3* | ++++ | ++++ | +++ | ++ |  | | ++++ | | ++++ | ++++ | ++++ |  |
| *Δclr4 Δiec3* | ++++ | + | - | - |  | | ++++ | | ++++ | +++ | +++ |  |

The extent of proliferation for each strain in Figure 3 was represented by: -, no growth, and increasing growth +, ++, +++, ++++.
